# Diagnostic and prognostic value of ^99m^Tc-MAA SPECT/CT for treatment planning of ^90^Y-resin microsphere radioembolization for hepatocellular carcinoma: comparison with planar image

**DOI:** 10.1038/s41598-021-82887-w

**Published:** 2021-02-05

**Authors:** Mai Hong Son, Le Ngoc Ha, Mai Hong Bang, Sungwoo Bae, Dinh Truong Giang, Nguyen Tien Thinh, Jin Chul Paeng

**Affiliations:** 1 Department of Nuclear Medicine, 108 Military Central Hospital, Hanoi, Vietnam; 2 Department of Hepato-Pancreato-Biliary disease, 108 Central Military Hospital, Hanoi, Vietnam; 3 Department of Nuclear Medicine, Seoul National University Hospital, 101 Daehak-ro, Jongno-gu, Seoul, 03080 Korea; 4 Department of Molecular Medicine and Biopharmaceutical Sciences, Graduate School of Convergence Science and Technology, Seoul National University, Seoul, Korea

**Keywords:** Diseases, Gastroenterology, Medical research, Oncology

## Abstract

^99m^Tc-macroaggregated albumin (MAA) imaging is performed before transarterial radioembolization (TARE), in which SPECT/CT is presumed more precise than planar image. However, additive role of SPECT/CT has not been well established. Thirty-four consecutive hepatocellular carcinoma patients of intermediate and advanced stages who underwent ^90^Y-microsphere TARE were recruited. On pre-treatment planning scan using ^99m^Tc-MAA, image characteristics and absorbed dose for target tumors calculated by partition model methods were estimated on planar image and SPECT/CT, respectively. The measurements were repeated on post-treatment ^90^Y PET/CT, as the reference standard. Treatment response was assessed and predictive values of image parameters were analyzed. The image characteristics including heterogeneity, necrosis and thrombosis uptake were better delineated on SPECT/CT than planar scan. The agreement and correlation of TNr between SPECT/CT and PET/CT were stronger than those between planar scan and PET/CT. Tumor dose estimated on ^99m^Tc-MAA SPECT/CT was more effective than planar image for prediction of treatment response, with cutoff value 125 Gy (sensitivity of 86% and specificity of 75%). In conclusion, ^99m^Tc-MAA SPECT/CT is more closely correlated with post-treatment ^90^Y PET/CT, and is more effective for predicting treatment response than planar scan. SPECT/CT is superior to planar image in simulation before ^90^Y TARE.

## Introduction

Hepatocellular carcinoma (HCC) is one of the most common causes of cancer-related mortalities in Asia Pacific^1^. Transarterial radioembolization (TARE) using ^90^Y-loaded resin or glass microsphere is an optional treatment for unresectable HCC with promising outcome^2,3^. TARE using ^90^Y-loaded resin microsphere is commonly preceded by transarterial perfusion scintigraphy using ^99m^Tc-macroaggregated albumin (MAA) for treatment planning. The goal of TARE is to deliver an effective radiation dose to the tumor while sparing normal liver. Treatment planning using ^99m^Tc-MAA imaging is based on liver-lung shunt fraction and tumor-to-normal liver uptake ratio, which are key factors for calculating radiation doses to the tumor, liver and lung^4^. The partition model with ^99m^Tc-MAA planar image is a well-established method for estimating radiation to the targeted tumor^5^.

However, delineation of the targeted tumor volume on the planar image is often not accurate. ^99m^Tc-MAA single-photon emission computed tomography (SPECT)/computed tomography (CT) is currently recommended instead of the planar image, because it has advantages over the planar image, such as accurate delineation of tumor margin and precise measurement of the delivered radioactivity^6^. Meanwhile, it has been reported that the dosimetry using ^99m^Tc-MAA imaging is different from that of post-treatment ^90^Y positron emission tomography (PET)/CT, which is considered as the gold standard^7,8^. Therefore, the simulation results with ^99m^Tc-MAA SPECT/CT should be validated with post-treatment ^90^Y PET/CT and compared with ^99m^Tc-MAA planar image. Garin et al. highlighted that dosimetry using ^99m^Tc-MAA SPECT/CT for simulation can predict tumor response and overall survival of HCC patients treated with glass microspheres^9^. Moreover, other studies reported that ^99m^Tc-MAA SPECT/CT has a good correlation with ^90^Y PET/CT in terms of tumor-to-normal liver uptake ratio and SPET/CT is valuable for detecting extrahepatic uptake for stratifying radiation injury risks to normal organs^10,11^.

To the best of our knowledge, no studies have been conducted on the role of ^99m^Tc-MAA SPECT/CT parameters in comparison with ^99m^Tc-MAA planar scan. Thus, this study aims to evaluate the accuracy of dosimetry using ^99m^Tc-MAA SPECT/CT and identify the role of SPECT/CT in the prediction of treatment response to TARE.

## Methods

### Patients

Consecutive HCC patients who underwent TARE in Hospital 108 from May 2017 to December 2019 were prospectively enrolled in the study. TARE indication was decided by the Liver Cancer Tumor Board of Hospital 108. A patient is deemed eligible for TARE in case of intermediate- or advanced-stage HCC, preserved hepatic function (Child–Pugh A), and Eastern Cooperative Oncology Group performance status 0–2. However, patients with main portal branch thrombosis, hepatic cirrhosis with Child–Pugh C, or lung shunt fraction of > 20% were excluded. The study design was approved by the Institutional Review Board of Hospital 108, and informed consent was obtained from each patient before being included in the study. All study procedures were performed following relevant guidelines and regulations.

### Simulation image acquisition and analysis

A contrast-enhanced abdominal CT scan was performed before simulation angiography for angiographic mapping and measuring the volumes of the normal liver and targeted tumor. Simulation diagnostic angiography was conducted for arterial mapping and selection of optimal catheter position for TARE. After selecting the tumor-feeding artery, ^99m^Tc-MAA (185 MBq) was injected into the selected artery. All the corresponding arteries were chosen for injection of ^99m^Tc-MAA and planned as target arteries for a tumor supplied by ≥ 2 feeding arteries.

Planar and SPECT/CT images were obtained using a dual-head gamma camera equipped with low-energy high-resolution collimators and 4-slice CT (Optima NM/CT 640, GE Healthcare, Milwaukee, WI, USA) 1 h after ^99m^Tc-MAA injection. In the planar scan, the field-of-view of ^99m^Tc-MAA scan covered the whole lungs and abdomen, and anterior/posterior images were acquired. A low-dose CT image was acquired (120 kVp, 30 mAs) and reconstructed with a slice thickness of 5 mm. For SPECT, 60 projection images with a 6° rotation angle were acquired using step-and-shoot mode for 12 s per frame. SPECT image was reconstructed on 128 × 128 matrices, using an ordered subset expectation maximization (OSEM; 2 iterations, 10 subsets) and the Butterworth filter (frequency 0.48, power 10). Attenuation correction was applied with the CT image.

Images were analyzed using a vendor-supplied analysis package (Xeleris 4.0, GE Healthcare, Milwaukee, WI, USA). On planar images, regions-of-interests (ROIs) were drawn for both lungs, whole liver and targeted tumor in the liver. Also on SPECT/CT image, ROIs were drawn for both lungs, liver and tumor on every axial slice using Dosimetry toolkit (GE Healthcare) with reference to angiography and contrast-enhanced CT. The ROIs were also used for measuring the tumor and liver volumes. The ROIs of the lungs were drawn with a sufficient margin from the liver, to prevent possible spillover activity. The necrotic part of a tumor was excluded from the analysis. Total counts of each ROI on either planar and SPECT/CT image were used to estimate lung shunt fraction (%LSF) and the ratio of the tumor-to-normal liver (TNr) according to the formula described on the European Association of Nuclear Medicine guidelines^12^. For a tumor supplied by ≥ 2 feeding arteries, the TNr of every tumor part supplied by each feeding artery and mean TNr of the whole tumor were estimated. Briefly, each artery-specific ROI was drawn on ^99m^Tc-MAA SPECT/CT based on boundaries of uptake on angiography and contrast-enhanced CT (Supplementary Fig. S1). The ROIs were also used for measuring the tumor volume of each feeding artery. The partition model was applied to estimate the mean absorbed doses of the lung (D_lung_), normal liver (D_liver_) and target tumor (D_tumor_). Injected radioactivity was planned so that D_tumor_ should be ≥ 120 Gy, D_lung_ < 20 Gy, and D_liver_ < 30 Gy^4,12^. Super-selective treatment based on the partition model was performed for a tumor located in the segment or supplied by ≥ 2 feeding arteries. The D_liver_ and D_tumor_ were selectively estimated with the corresponding tumor and normal liver.

### Treatment and response evaluation

After dosimetry for targeted tumor, ^90^Y-resin microspheres (SIR-Sphere, SIRTeX™, Sydney, Australia) of calculated dose were injected to tumor-supplying arteries. Briefly, the catheter tip was placed at the same position as that of the ^99m^Tc-MAA injections, and the prescribed ^90^Y-resin microsphere activity was slowly and gently injected to prevent reflux, through the vendor-supplied delivery system. The net injected dose was within the expected range, as previously reported^13^. Post-treatment PET/CT scan was performed with the protocol described in a previous study^14^, using a dedicated PET/CT scanner (GE Discovery 710, GE Healthcare) after 6 h. A CT scan was performed first (140 kVp, dose modulation 30–300 mA, 3.75-mm slice thickness) and the PET image was obtained for 3 bed positions (15 min per bed position) and reconstructed using a 3D OSEM algorithm (3 iterations, 18 subsets) integrated with the time-of-flight and point-spread function recovery.

The image characteristics including the pattern of tumor uptake, necrosis, and thrombosis on each ^99m^Tc-MAA planar image, SPECT/CT and post-treatment ^90^Y PET/CT were interpreted at different time point for each modality by two experienced nuclear medicine physicians, and a consensus was made. On ^90^Y PET/CT images, ROIs were drawn for the lungs, liver, and targeted tumors with the same method used for SPECT/CT, to estimate the %LSF and TNr. The TNr values were compared between image modalities.

Treatment response was evaluated on follow-up contrast-enhanced CT obtained at approximately 3 months after treatment using the modified RECIST criteria^15^. A patient was classified as a responder in case of complete or partial remission, and as a non-responder in case of stable disease or progressive disease, respectively.

### Statistical analysis

Commercial software packages were used for statistical analysis (SPSS v.20.0, IBM Corp.; GraphPad Prism v8.0, GraphPad Software Inc.). Pearson’s correlation coefficient and Bland–Altman plot were used for evaluating the correlation and agreement of %LSF and TNr values. Cohen’s kappa coefficient was used to measure the agreement between image characteristics of planar, SPECT/CT, and PET/CT. Categorical values were compared using the chi-square test or Fisher’s exact test. Continuous variables with normal distribution were compared using paired Student *t*-test or repeated measure ANOVA. Variables not following normal distribution were compared using Mann–Whitney test. The optimal cutoff value, sensitivity, and specificity for treatment response prediction were calculated using receiver-operating characteristic (ROC) curve analysis, and diagnostic power was assessed using the area under the curve (AUC). Logistic regression analysis was used to determine the significant parameters to predict treatment response. The significance threshold was set at *P* < 0.05.

## Results

### Patients and TARE

A total of 34 patients (M:F = 32:2; age 53.8 ± 14.2 years) were included in the study, and 45 targeted lesions were treated with TARE. The tumor size was 753 ± 631 mL (range 75–1680 mL). The mean radioactivity of the injected ^90^Y-resin microsphere was 2.16 ± 0.99 GBq. Eleven (32%) patients were treated for two target supplying arteries. The tumor location was in the right lobe in 32 patients (94%), and left lobe in 2 patients (6%). Follow-up CT scans were performed at 2.8 ± 0.8 months after TARE. The responders and non-responders were 22 (64.7%) and 12 (35.3%), respectively. Table 1 summarizes the patient characteristics and treatment details.

**Table 1:** General characteristic of patients.

Variables	Values
Age (years)	53.8 ± 14.2
**Gender**	
Male	32 (94%)
Female	2 (6%)
**Underlying disease**	
Hepatitis B	33 (97%)
Hepatitis C	0 (0%)
**Tumor location**	
Right lobe	32 (94%)
Left lobe	2 (6%)
Tumor volume (mL)	752.9 ± 630.9 (range, 75–1680)
Portal vein branch thrombosis	18/34 (52.9%)
Tumor necrosis	12/34 (35.3%)
**Barcelona stage**	
Intermediate	6/34 (17.6%)
Advanced	28/34 (82.4%)
Number of targeted lesions	45
Radioactivity of ^90^Y (GBq)	2.16 ± 0.99
**Target supplying artery**	
1	23/34 (67.6%)
2	11/34 (32.4%)
**Treatment response**	
Responder	22/34 (64.7%)
Non-responder	12/34 (35.3%)

### Image characteristics of ^99m^Tc-MAA planar scan, SPECT/CT and ^90^Y PET/CT

The patterns of tumor uptake including heterogeneity, necrosis and thrombosis uptake were well delineated on SPECT/CT, which showed stronger agreement with those on ^90^Y PET/CT than those on the planar image (Table 2). The image characteristics of SPECT/CT were concordant with those of ^90^Y PET/CT (Supplementary Fig. S2), although tumor thrombosis uptake was more readily found on PET/CT. Both of planar image and SPECT/CT did not show any uptake in extrahepatic organs such as the digestive organs.

**Table 2:** Image characteristics and measurements of ^99m^Tc-MAA planar image, SPECT/CT and ^90^Y PET/CT.

Variables	Planar	SPECT/CT	PET/CT	Kappa value	
				Planar vs. PET/CT	SPECT/CT vs. PET/CT
Heterogeneity	14/34 (41.2%)	20/34 (58.8%)	20/34 (58.8%)	0.254	0.843
Necrotic tumor	4/34 (11.7%)	12/34 (35.3%)	12/34 (35.3%)	0.353	0.706
Thrombosis uptake	0 (0%)	4/34 (11.7%)	10/34 (29.4%)	0.000	0.380
Extrahepatic uptake	0 (0%)	0 (0%)	0 (0%)	1.000	1.000

**Figure 1:**
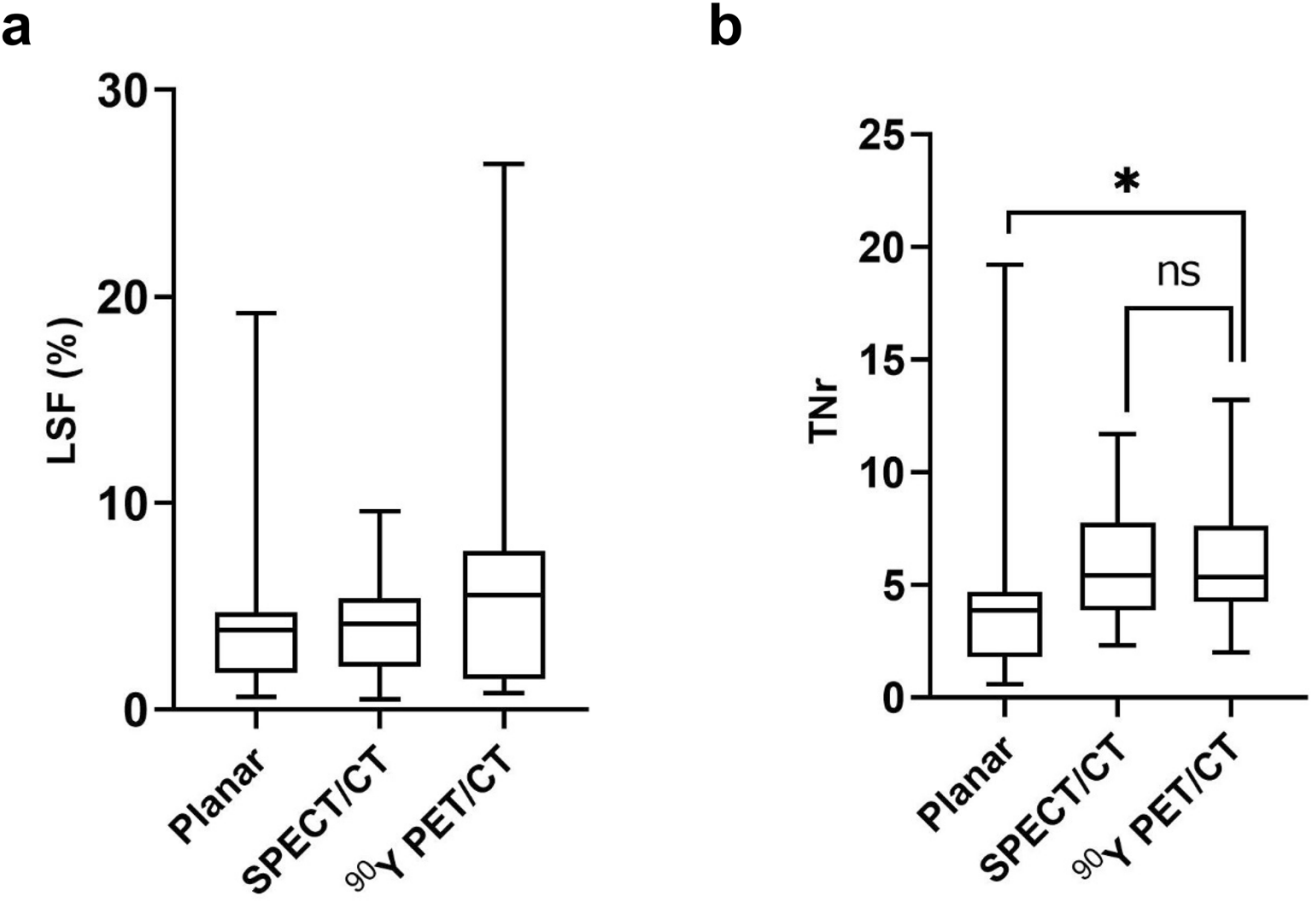
Comparison of %LSF (**a**) and TNr (**b**) between Planar, SPECT/CT and PET/CT.

**Figure 2:**
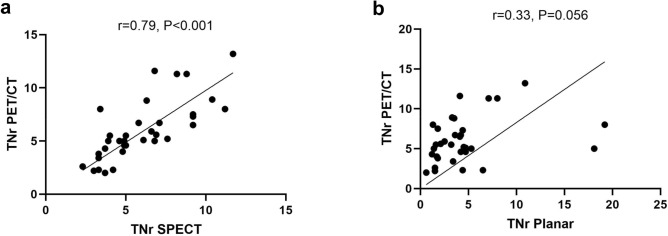
Correlation of TNr between different images. Correlation coefficient between SPECT/CT and PET/CT (**a**) was higher than that between planar image and PET/CT (**b**).

**Figure 3:**
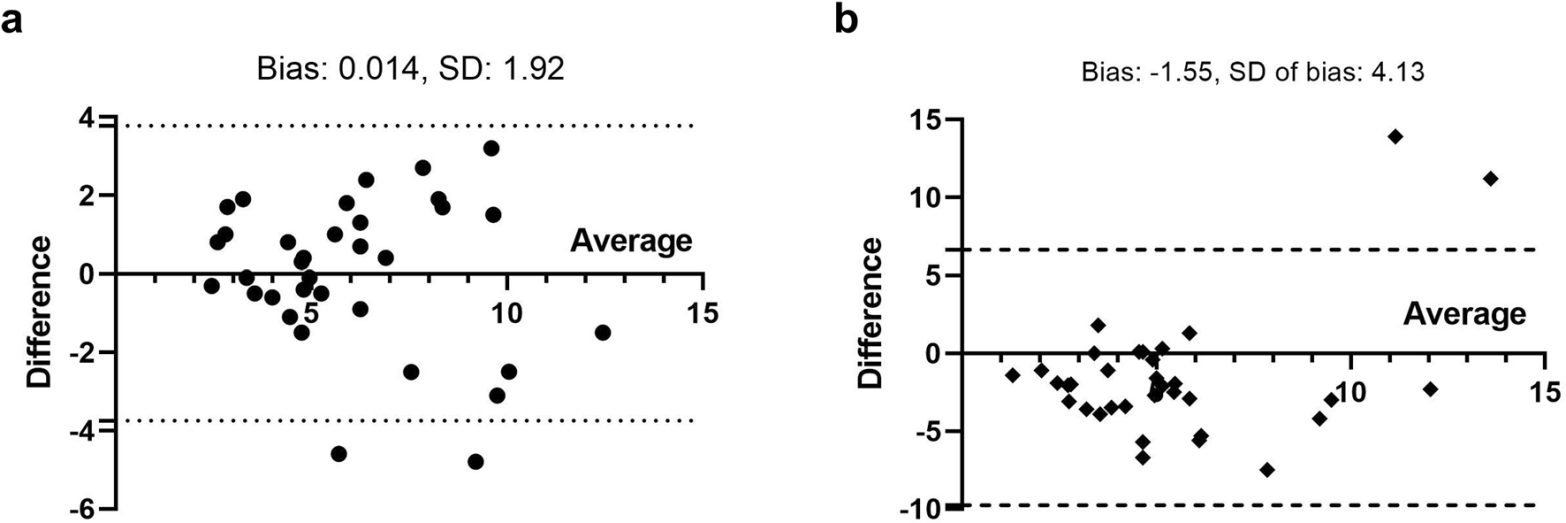
Bland–Altman plots for agreement of TNr between SPECT/CT and PET/CT (**a**); between planar image and PET/CT (**b**).

%LSFs were 3.85 (range 0.6–19.2), 4.15 (range 0.5–9.6), and 5.55 (range 0.8–26.4) on planar image, SPECT/CT, and PET/CT, respectively. No statistically significant difference was noted in %LSF between planar and PET/CT (*P* = 0.501), or between SPECT/CT and PET/CT (*P* = 0.999; Fig. 1a). However, there was a significant difference in TNr between planar image and PET/CT (*P* < 0.001), whereas no significant difference was noted between SPECT/CT and PET/CT (*P* = 0.999; Fig. 1b). The correlation coefficient of TNr between SPECT/CT and PET/CT was higher than that between planar image and PET/CT (*r* = 0.79, *P* < 0.001 *vs*. *r* = 0.33, *P* = 0.056; Fig. 2). In the Bland–Altman plot, the agreement of TNr between SPECT/CT and PET/CT was also higher than that between planar image and PET/CT (Fig. 3).

### Predictive value of ^99m^Tc-MAA scan and SPECT/CT for treatment response

**Figure 4:**
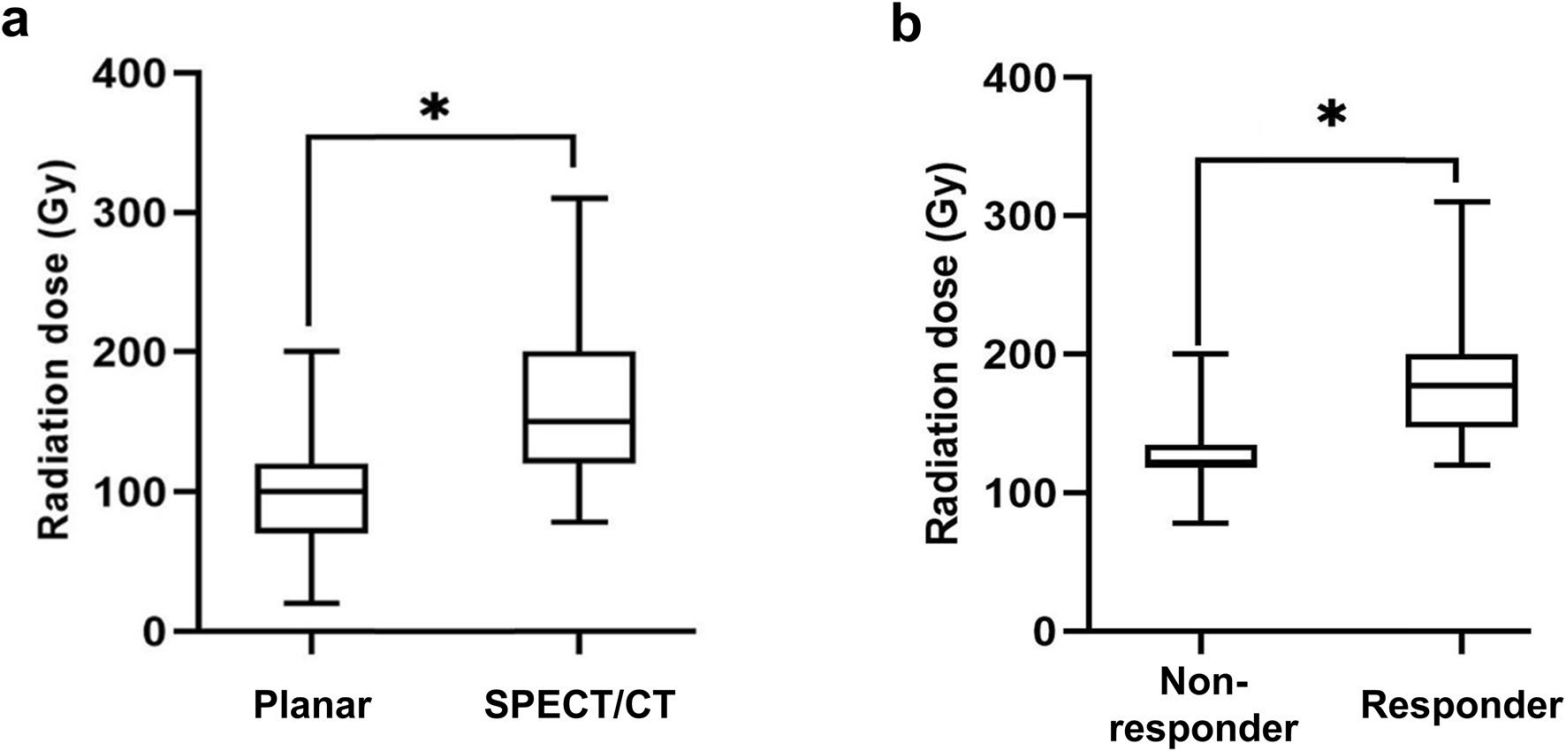
D_tumor_ values measured on planar image and SPECT/CT. (**a**) D_tumor_ was significantly higher on SPECT/CT than on planar image. (**b**) D_tumor_ of responder was significantly higher than that of non-responder.

**Figure 5:**
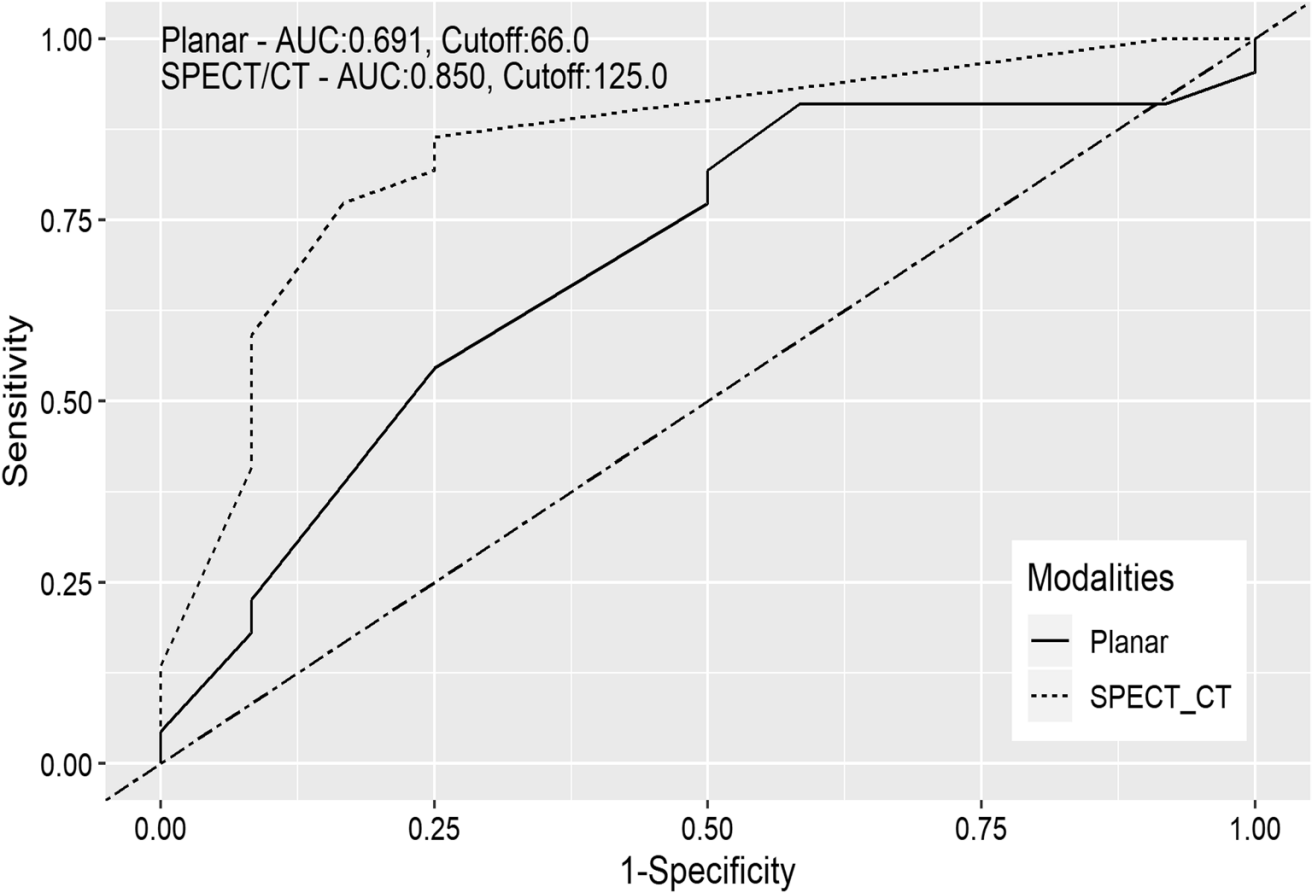
ROC curves of D_tumor_ values in predicting treatment response. AUC of SPECT/CT (dashed line) was significantly higher than that of planar image (continuous line) (*P* = 0.023).

**Figure 6:**
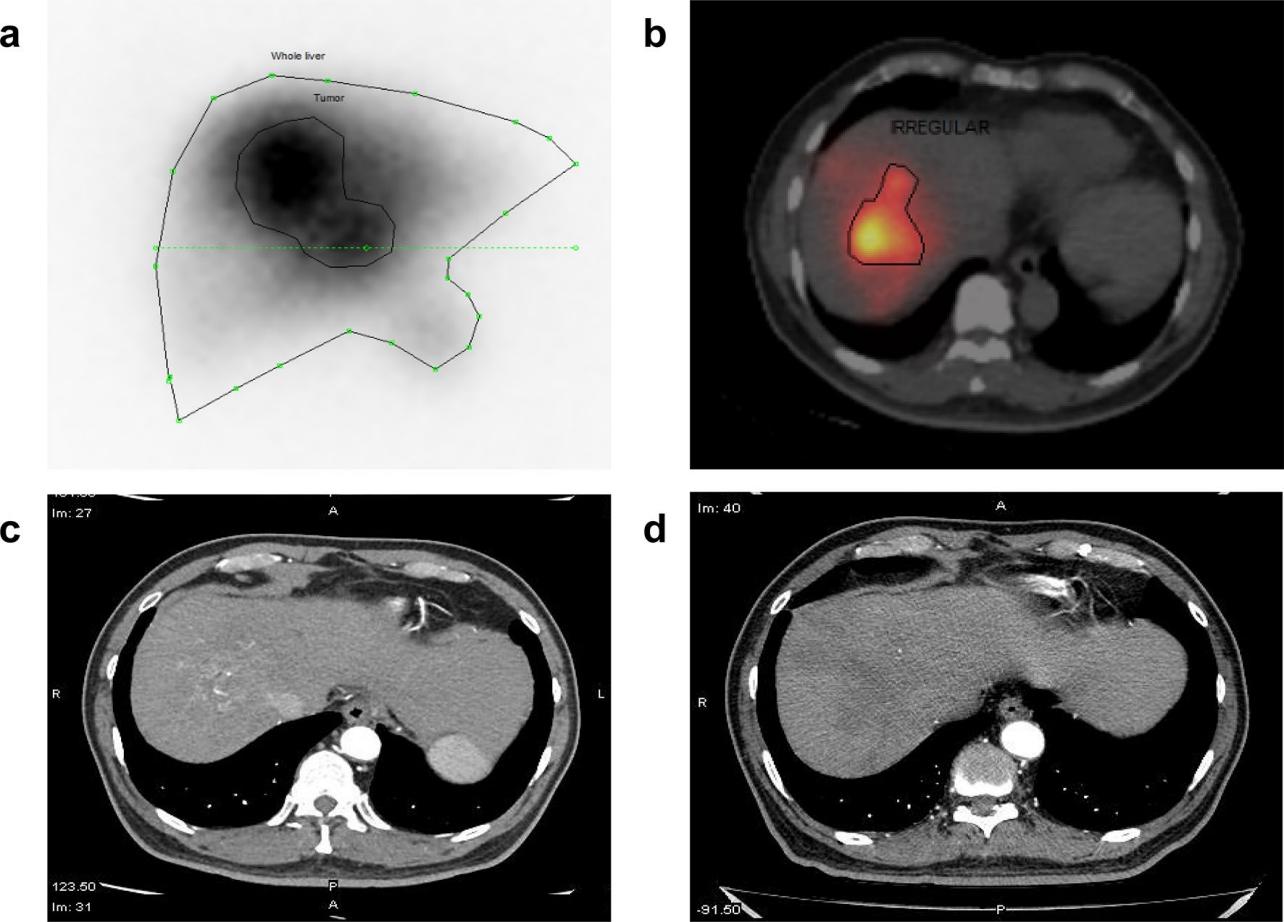
Images of a representative case. A 70-year-old male diagnosed with advanced stage of HCC, underwent ^99m^Tc-MAA planar scan (**a**) and SPECT/CT (**b**). Planar image showed only heterogeneous tumor uptake in the right liver, whereas SPECT/CT depicted better tumor margin by delineating viable tumor with excluding necrotic part (**b**). In comparison with CT before treatment (**c**), complete treatment response was seen on follow-up CT after 2 months (**d**).

Median D_tumor_ on SPECT/CT was 150 Gy (range 78–310 Gy), which was significantly higher than that on the planar images, 100 Gy (range 20–200 Gy; *P* < 0.001, Fig. 4a). The D_tumor_ of the responders on SPECT/CT was significantly higher than that of the non-responders (median 178 Gy, range 120–310 Gy *vs*. median 120 Gy, range 78–200 Gy; *P* < 0.001; Fig. 4b). In the ROC curve analysis for response prediction, AUC of D_tumor_ on SPECT/CT was 0.85 (95% CI, 0.71–0.99; *P* = 0.001), which was higher than that on the planar scan (0.691, 95% CI, 0.5–0.88; *P* = 0.069). The difference in the AUC values was statistically significant (*P* = 0.023). In the ROC curve analysis, the optimal cutoff value of D_tumor_ on SPECT/CT for predicting treatment response was 125 Gy. With this cutoff value, the sensitivity and specificity were 86% and 75%, respectively (Fig. 5). In logistic regression analysis, the volume of tumor uptake, TNr and D_tumor_ on SPECT/CT were significant factors for predicting treatment response (Table 3). A representative case is shown in Fig. 6.

**Table 3:** Logistic regression analysis for risk factors of treatment response prediction.

Factors	Relative risk (95% CI)	*P*
Heterogeneity of uptake	1.032 (0.247–4.303)	0.966
Tumor necrosis	0.375 (0.086–1.631)	0.191
Portal vein thrombosis uptake	0.500 (0.061–4.091)	0.518
Volume of tumor on SPECT/CT	1.004 (1.001–1.007)	0.007
LSF on SPECT/CT	0.919 (0.677–1.248)	0.589
TNr on SPECT/CT	0.627 (0.415–0.948)	0.027
D_tumor_ on SPECT/CT	0.956 (0.923–0.989)	0.011

## Discussion

The key finding of this study is that ^99m^Tc-MAA SPECT/CT has higher diagnostic and prognostic values compared with the planar scan. Consequently, SPECT/CT showed stronger agreement with post-treatment ^90^Y PET/CT compared with the planar scan. Furthermore, dosimetry estimated on SPECT/CT showed higher sensitivity and specificity for predicting treatment response than those of planar scan.

^99m^Tc-MAA distribution on pre-treatment planning scan is considered as a surrogate marker for radiation dose in TARE using ^90^Y-microspheres^16^. Generally, angiographic injection of ^99m^Tc-MAA and planar and/or SPECT scan are routinely used before TARE^17–20^. ^99m^Tc-MAA imaging aims to map the tumor-feeding vessels and detect extrahepatic uptake, which can be a potential cause of complications due to the reflux of ^90^Y-microspheres^21,22^. Ahmadzadehfar et al. reported that ^99m^Tc-MAA SPECT/CT is more effective than the planar scan for identifying extrahepatic uptake with a sensitivity of 100% and a specificity of 93%, whereas that of planar scan is 32% and 98%, respectively^10^. Another study confirmed the superior diagnostic value of SPECT/CT in the detection of extrahepatic uptake in the digestive system^23^.

In this study, ^99m^Tc-MAA SPECT/CT was used as a guide for selective artery-based TARE and the partition model was used for dosimetry. Although the partition model is more laborious than the simple methods such as the body surface area method, it can provide more accurate information on tumor dose^6^. The body surface area method may overestimate the tumor radiation dose, and the real delivered dose is often lower than that in the partition model method^24^. Furthermore, dosimetry by SPECT/CT and the partition model has been reported to be effective in estimating radiation doses to tumor and normal liver in ^90^Y-microsphere TARE^6,25^. SPECT/CT-based dosimetry showed high concordance with post-treatment ^90^Y PET for both resin and glass microspheres^26^. Moreover, SPECT/CT is effective for the accurate delineation of tumor volume, exclusion of necrotic tumor portion, and detection of extrahepatic uptake which are the basis for precise tumor dosimetry. Planar image and/or SPECT alone has limited efficacy in achieving accurate measurement of tumor volume, and the parameters obtained from these images are often underestimated^27^. ^99m^Tc-MAA SPECT/CT is more appropriate in showing the functional status of a tumor, because it can show the masses of viable tumor, necrotic tumor, and non-tumorous liver better than SPECT alone or planar image. This study showed that heterogeneous tumor uptake and necrotic tumor are more often observed on SPECT/CT than on planar images. When compared with post-TARE ^90^Y PET/CT, ^99m^Tc-MAA SPECT/CT showed excellent concordances with PET/CT results, except for thrombosis uptake detection. SPECT/CT showed 4 in 34 cases with thrombosis uptake whereas PET/CT detected 14 in 34 cases, probably because of a difference of spatial resolution between ^99m^Tc-MAA SPECT/CT and ^90^Y-microspheres PET/CT.

The dosimetry results of SPECT/CT played a significant role in therapeutic planning^28^. In the partition model dosimetry, TNr and %LSF are the frameworks for efficacious planning dosimetry. Based on those values, ^90^Y-microsphere activity can be determined to deliver the maximum dose to target tumors while sparing normal organs. Although TNr < 2.0 could be considered as a safety margin for ^90^Y-microsphere administration on the planar scan, SPECT/CT may provide a more precise TNr measurement than the planar scan^29,30^. TNr estimated on SPECT/CT was more reliable than that on the planar image in this study. When ^90^Y PET/CT was used as a reference standard, SPECT/CT showed much stronger TNr agreement and correlation with PET/CT than the planar image. The discrete ROIs of targeted tumors can be defined more accurately by SPECT/CT than by planar scan, especially for multiple lesions^31^. The radiation doses for each tumor and patient can be estimated using the partition model by calculating the TNr for each tumor and the average TNr for a patient^31^.

The present study demonstrated that planar image underestimates the radiation dose to the tumor compared with SPECT/CT, and dosimetry with SPECT/CT is more accurate for treatment response evaluation. This study showed that the tumor radiation dose estimated by SPECT/CT is higher in responders than in non-responders, and that the best D_tumor_ cutoff value for response prediction is 125 Gy. In a similar population study including 42 patients, the best D_tumor_ threshold to predict treatment response is 61 Gy with a sensitivity and specificity of 75%^32^. Garin et al. reported that the radiation doses of the responders are higher than those of the non-responders after ^90^Y-glass microsphere treatment, and that the minimum radiation dose should be 205 Gy^33^. However, the cutoff values of the radiation doses to achieve treatment response have been discordant among published studies. It was highlighted that the absorbed dose of 120 Gy is recommended for resin microsphere, which was different from that of glass microsphere^4,6,16,34^. The treatment response after ^90^Y-glass microsphere may depend not only on the absorbed dose but also on microscopic heterogeneity of uptake, thrombosis, tumor size, and disease stage^33^. In this study, the volume of the viable tumor on SPECT/CT or TNr was another factor along with D_tumor_ for predicting treatment response after ^90^Y-resin microsphere.

The present study has several limitations. First, the prognostic role of image parameters on ^99m^Tc-MAA SPECT/CT was not assessed in terms of overall and progression-free survival for the unresectable HCC. It was challenging to follow up patients for long-term, because the hospital in this study is a tertiary referral center and many patients were referred for TARE from different regions or countries. Second, the associations of TNr, LSF and D_tumor_ with tumor uptake pattern, tumor size, and portal vein thrombosis presence were not analyzed in this study, because the patient number of each group was too small for statistical analysis.

In conclusion, this study highlights that ^99m^Tc-MAA SPECT/CT can be a more reliable simulation tool than the planar scan before ^90^Y TARE. The image parameters for dosimetry showed higher agreement and correlation between ^99m^Tc-MAA SPECT/CT and ^90^Y PET/CT, than those between planar scan and PET/CT. Radiation doses to targeted tumors estimated on ^99m^Tc-MAA SPECT/CT can predict treatment response in TARE using ^90^Y-microspheres. Thus, SPECT/CT is superior to planar imaging in simulation before ^90^Y TARE, and the use of SPECT/CT is strongly recommended.

## Supplementary Information

Supplementary Information.
