# Supplementary material for: Diagnostic and prognostic value of 99mTc-MAA SPECT/CT for treatment planning of 90Y-resin microsphere radioembolization for hepatocellular carcinoma: comparison with planar image
Source: Sci Rep. 2021 Feb 5;11:3207. doi: 10.1038/s41598-021-82887-w (PMC7864932; doi:10.1038/s41598-021-82887-w)
Supplement: Supplementary file 1 — Supplementary Information. [file 41598_2021_82887_MOESM1_ESM.pdf]

Title:

Diagnostic and Prognostic Value of ^{99m}Tc-MAA SPECT/CT for Treatment Planning of ^{90}Y-Resin Microsphere Radioembolization for Hepatocellular Carcinoma: Comparison with Planar Image

Authors

Mai Hong Son; Le Ngoc Ha; Mai Hong Bang; Sungwoo Bae; Dinh Truong Giang; Nguyen Tien Thinh; Jin Chul Paeng

Supplementary Figure 1

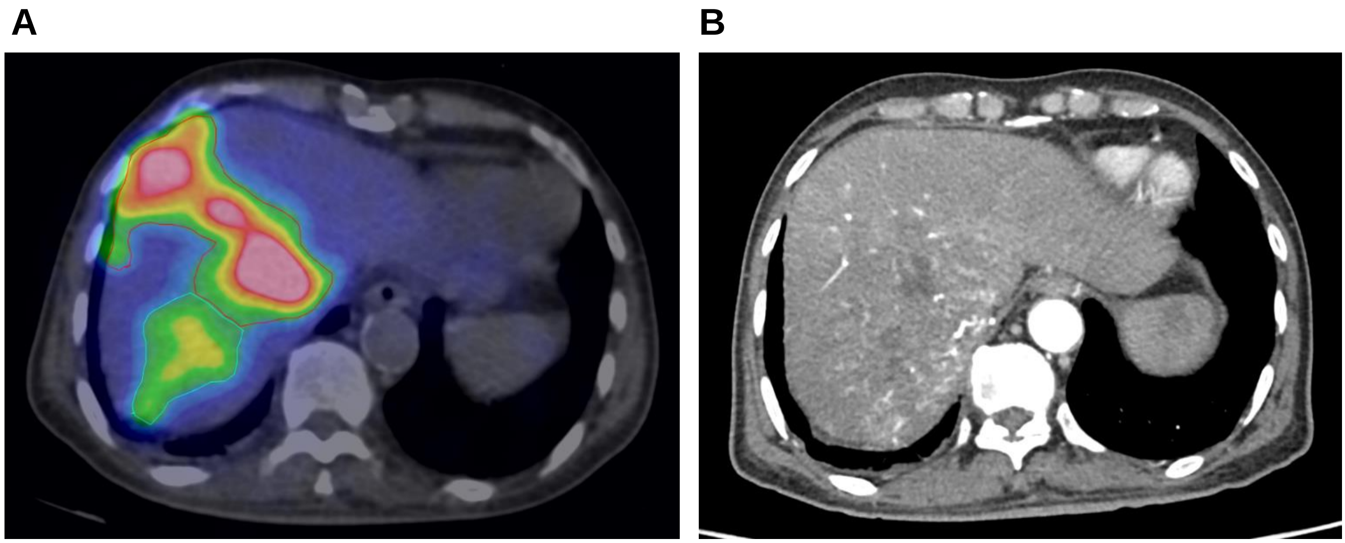

An example of drawing ROIs for each part of tumor supplied by dual feeding arteries. A 79-year-old male diagnosed with advanced stage of HCC, underwent ^{99m}Tc-MAA SPECT/CT (A) for treatment planning. (A) shows different artery-specific ROIs for tumor part in segment VIII, supplied by the anterior branch of hepatic artery (red) and for tumor part in segment VII supplied by the posterior branch of hepatic artery (green). The ROIs were drawn based on the highest uptake and border of uptake, with reference to contrast-enhanced CT (B). The adjacent normal liver was also delineated to measure the count. Volumes and counts of the each part of tumor, whole tumor and normal liver were measured and used for dosimetry.

Supplementary Figure 2

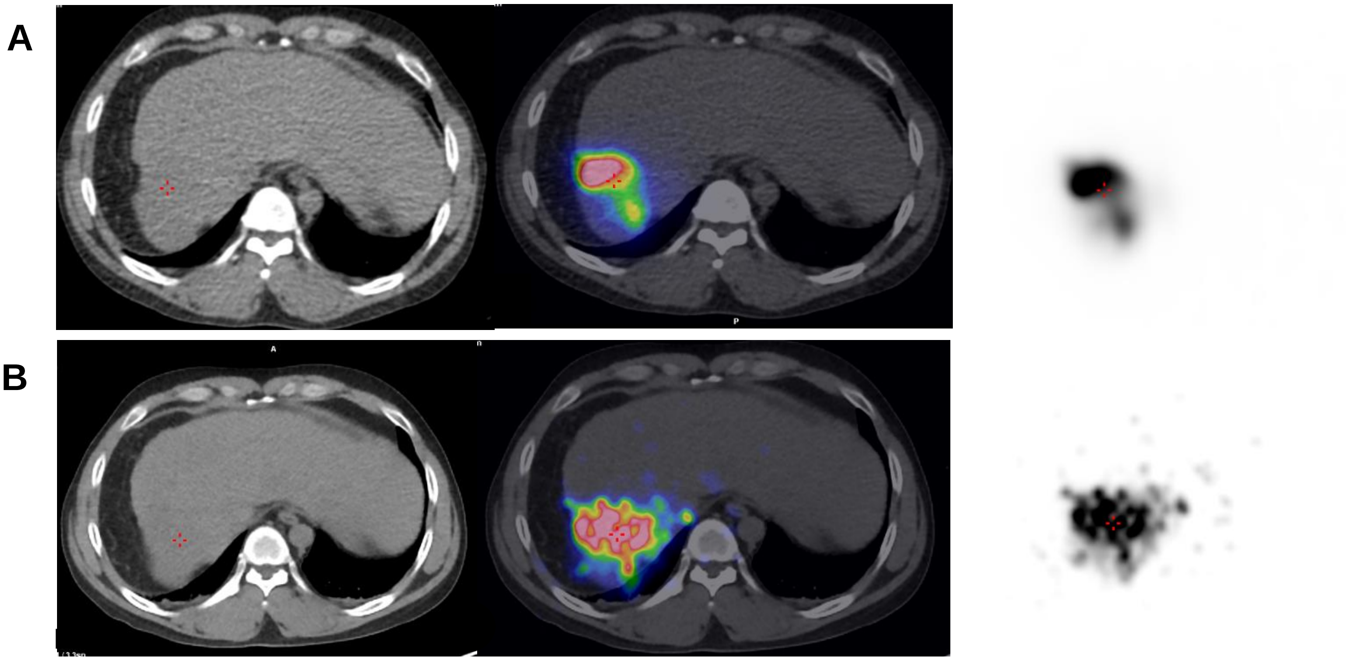

Images of a case for comparing ^{99m}Tc-MAA SPECT/CT (A) and ^{90}Y PET/CT (B)
